# Conservation of mRNA secondary structures may filter out mutations in *Escherichia coli* evolution

**DOI:** 10.1093/nar/gkt507

**Published:** 2013-06-19

**Authors:** Andrey Chursov, Dmitrij Frishman, Alexander Shneider

**Affiliations:** ^1^Department of Genome Oriented Bioinformatics, Technische Universität München, Wissenschaftzentrum Weihenstephan, Maximus-von-Imhof-Forum 3, D-85354, Freising, Germany, ^2^Helmholtz Center Munich—German Research Center for Environmental Health (GmbH), Institute of Bioinformatics and Systems Biology, Ingolstädter Landstraße 1, D-85764 Neuherberg, Germany and ^3^Cure Lab, Inc., 43 Rybury Hillway, Needham, MA 02492, USA

## Abstract

Recent reports indicate that mutations in viral genomes tend to preserve RNA secondary structure, and those mutations that disrupt secondary structural elements may reduce gene expression levels, thereby serving as a functional knockout. In this article, we explore the conservation of secondary structures of mRNA coding regions, a previously unknown factor in bacterial evolution, by comparing the structural consequences of mutations in essential and nonessential *Escherichia coli* genes accumulated over 40 000 generations in the course of the ‘long-term evolution experiment’. We monitored the extent to which mutations influence minimum free energy (MFE) values, assuming that a substantial change in MFE is indicative of structural perturbation. Our principal finding is that purifying selection tends to eliminate those mutations in essential genes that lead to greater changes of MFE values and, therefore, may be more disruptive for the corresponding mRNA secondary structures. This effect implies that synonymous mutations disrupting mRNA secondary structures may directly affect the fitness of the organism. These results demonstrate that the need to maintain intact mRNA structures imposes additional evolutionary constraints on bacterial genomes, which go beyond preservation of structure and function of the encoded proteins.

## INTRODUCTION

Increasing experimental (1) and computational (2,3) evidence points to the existence of extensive RNA structures in the coding regions of mRNA molecules. RNA secondary structures have been implicated in regulation of translation initiation, elongation and termination in both prokaryotes and eukaryotes (4,5). In particular, the anti-correlation between translation efficiency and the thermodynamic stability of local secondary structure in the vicinity of the translation initiation site has been thoroughly documented (6). RNA hairpins are thought to be involved in controlling mRNA decay (7), localization (8–10) and interaction with other molecules (11). Overall, the mRNA coding regions appear to be more structured than the untranslated regions (1) and have lower minimum folding free energies. Hence, the mRNA coding regions appear to have more stable structures than codon-randomized sequences (12). Owing to the need to simultaneously preserve both the function and structure of the encoded protein, as well as the structural elements of the RNA molecule itself, mRNA coding regions are subject to dual selection pressure.

Using a mammalian system, we have recently shown that mutations altering secondary structures of influenza mRNAs may serve as a functional knockout of the corresponding genes (13). More recently, Moss *et al.* (14) established a direct connection between mutation patterns in the influenza virus genome and the hydrogen-bonding patterns shaping RNA structures. Thus, preservation of viral RNA structures and elimination of mutations disruptive for RNA structures may be a previously unknown mechanism of viral evolution. In the present article, we put forward the hypothesis that conservation of RNA structures may also play a role in bacterial evolution. To examine this hypothesis, we compared the genomes of parental and progeny *Escherichia coli* clones standing 40 000 generations apart. The ‘long-term evolution experiment’ (15–18) tracking genetic changes in 12 populations of *E. coli* was started by Richard Lenski in February 1988. All 12 replicate populations have originated from a single cell of the baseline strain, which was an *E. coli* B clone, and have been propagated at 37°C in liquid culture. Every 500 generations, samples for each population were frozen away at −80°C and retained for sequencing and comparison with their predecessors.

If our hypothesis is correct, mutations in essential genes that disrupt mRNA secondary structures would lead to insufficient gene expression and, due to the essentiality of those genes, such mutations would be filtered out as lethal. By contrast, selection against mutations disrupting mRNA secondary structures of nonessential genes would be expected to be less pronounced because an altered expression level of nonessential genes would not influence bacterial propagation. Supplementary Figure S1 exemplifies predicted structural perturbations induced by mutations altering minimal free energy of an *E. coli* mRNA.

To demonstrate this effect, one would ideally need to calculate exact secondary structures for both the original and the mutated mRNAs, compare them and make an inference about the changes in the RNA structure caused by mutations. However, a single RNA molecule may fold into more than one conformation (19,20). With increasing sequence length, the number of possible structures that an RNA molecule can adopt with similar (in many cases even the same) values of folding energy increases as well (21), thereby resulting in diminished prediction accuracy. Another well-known complication is that predicting secondary structures with pseudoknots is an NP-hard problem (22), which necessitates using approximations in structure prediction algorithms. Therefore, instead of calculating explicit secondary structure shapes for mRNAs, we pursued an indirect method of assessing whether mutation(s) affect secondary structures by quantifying minimum free energy (MFE) change. While different RNA structures may have exactly the same MFE, different MFE values are guaranteed to correspond to different structures. Despite the fact that a mutation did not change MFE does not mean that the RNA structure remained the same, yet, an opposite situation is reliably conclusive. The more mutations change the MFE, the greater affect on a secondary RNA structure they have.

Using this approach, we investigated how mutations observed in essential and nonessential genes influence the MFE values of mRNA structures. This article presents evidence that mutations in essential genes of *E. coli* that occurred during the ‘long-term evolution experiment’ changed the MFE of mRNA secondary structures to a lesser extent than mutations in nonessential genes. We emphasize that we focus exclusively on the conservation of secondary structures of mRNA coding regions and do not consider noncoding RNAs. This finding supports our hypothesis that mutations disrupting the mRNA structure of essential genes are filtered out during the course of bacterial evolution.

## MATERIALS AND METHODS

### Experimental data on evolutionary mutations in *E. coli*

In our analysis, we used data on genetic polymorphisms in *E. coli* accumulated in the course of the ‘long-term evolution experiment’ (16–18). Specifically, mutations in the 40 000th generation of one of the populations (Ara-1), with the ancestral strain REL606 (GenBank accession number NC_012967.1), were investigated. In this 40 000th clone, 627 single-nucleotide polymorphisms (SNPs) and 26 deletions, insertions and other polymorphisms were detected. Hereinafter, we take into account only SNPs. Ninety-two mutations occurring in intergenic regions as well as six mutations in pseudogenes and one mutation in an insertion sequence element were excluded from consideration. We also ignored one SNP owing to an inconsistency between the mutated nucleic acid, as reported in (18) and the nucleic acid occurring at this position in the complete genome sequence. Two genes with available SNP data were not considered: one with an inconsistency between its nucleotide and amino acid sequences, and another that had one of the reported mutations in its start codon. Our final data set contained 523 mutations involving 485 genes.

### Data on essential and nonessential genes of *E. coli*

There is no essentiality data available for the B strain of *E. coli*, but it is closely related to the well-studied *E. coli* K-12 MG1655 (23,24). For this latter strain (GenBank accession number U00096.2) Gerdes *et al.* (25) experimentally identified 620 genes as essential and 3126 genes as dispensable using a genetic footprinting technique. Because of the numerous discrepancies between the gene names, we conducted similarity-based transfer of essentiality data from the MG1655 strain to the REL606 strain, using the bidirectional best hit strategy to identify orthologous genes. Using blastp (26), we aligned all mutated genes from the REL606 genome against all genes from the MG1655 genome and *vice versa*. Genes from the two genomes were considered orthologous if they were the best hits for each other, with amino acid sequence identity >75% and e-value <10^−^^25^. This procedure enabled us to map 456 out of the 485 mutated genes in REL606 to the MG1655 strain, of which 48 were essential, 348 dispensable and 60 had undefined essentiality according to the MG1655 annotation.

### MFE values of RNA secondary structures

For each of the 48 essential and 348 nonessential mutated genes, we calculated MFE values of secondary RNA structures for both the original ancestral mRNAs and their 40 000th generation counterparts. We used the RNAfold tool from the Vienna RNA Package with the command line option noLP, which disallowed base pairs that can only occur as helices of length 1 (27).

### Generation of randomly mutated mRNAs

For each gene reported in (18) as possessing mutation(s) in the 40 000th generation, we produced an *in silico* family of random counterparts. Synonymous random mutations were introduced into ancestral mRNAs. When compared with the ancestral strain, each computer-generated RNA sequence had the same number of point mutations as the respective 40 000th generation mutant. There are six types of possible nucleotide substitutions: C:G → A:U; A:U → C:G; A:U → U:A; C:G → G:C; C:G → U:A; A:U → G:C. We introduced random mutations in such a way that the frequency for any given nucleotide substitution type was similar for essential and nonessential gene groups (Supplementary Table S1) and free of transition to transversion bias. For each gene, the ratio of transitions to transversions was calculated, and distributions of these ratios were compared for essential and nonessential genes. According to the Mann–Whitney U test, these distributions do not differ (*P* = 0.38). For the purpose of this work, we did not have to simulate *in silico* relative frequencies of nucleotide substitution observed reported by Wielgoss *et al.* (28). The MFE of secondary RNA structure was calculated for each computer-generated sequence by the RNAfold tool as described above.

**Figure 1.**
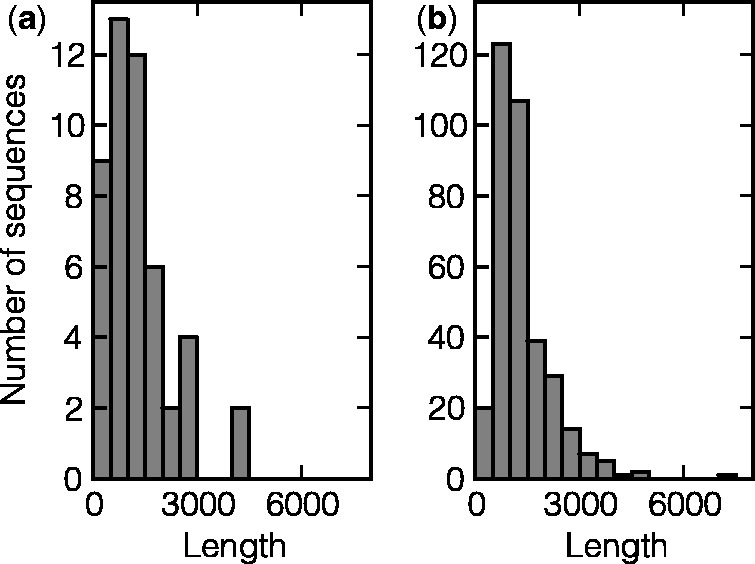
Histograms of gene lengths for essential (**a**) and nonessential (**b**) genes.

The number of computer-generated sequences varied from gene to gene dependent on gene length (Figure 1). If a short gene possesses only one nucleotide substitution *in vitro*, the number of conceivable *in silico* generated sequences having only one nucleotide changed is limited to an exhaustive set of synonymous point mutations (e.g. 516 variants for the gene *yciT* of length 750). For sufficiently long genes (typically >1300 bases), the subset of 1000 sequences with randomly introduced SNPs was used for further analysis.

### Statistical test

To find out whether *in vitro* mutations in essential and nonessential genes differ in their affect on MFE and mRNA secondary structures, we applied the following analysis. First, for each gene, we determined the absolute value of the difference between the MFE of the ancestor RNA and the MFE of the *in vitro* mutant, as well as that of each of the computer-generated mutants. Then, we calculated the fraction of computer-generated mutants whose absolute values of MFE differences were lower than the corresponding *in vitro* mutant. Each gene in the data set of essential genes and in the data set of dispensable genes was thus characterized by a percentile value. The Mann–Whitney U test was then used with the null hypothesis (H_0_) that the percentile values for essential and nonessential genes are from the same distribution.

### Data availability

The lists of defined essential and nonessential genes with the corresponding MFE values are presented in the Supplementary Tables S2 and S3, respectively.

## RESULTS

The main scientific questions we addressed in this study are whether purifying selection tends to eliminate mutations that are disruptive for mRNA structures, and whether this effect is more pronounced in essential genes compared with dispensable ones. Our methodology involved a comparison of actual mutations observed in the 40 000th generation of the ‘long-term evolution experiment’ (18) with a pool of random computer-generated mutations.

As an example, for a gene harboring two point mutations, we generated a thousand *in silico* mutants with two mutations each. MFE was calculated for the ancestor mRNA as well as for the mRNA of the 40 000th generation mutant experimentally observed in a Petri dish and for those of the *in silico* mutants. Owing to slight sequence changes, the RNA folding energies of both experimentally recorded and computer-generated mutants will be somewhat different from the MFE of the ancestor’s mRNA. We calculated the fraction of *in silico* mutants with a lesser extent of MFE change than the mutant observed *in vitro*. Suppose, for example, that the MFE of the ancestor mRNA was −5 kcal/mol and that the MFE of the *in vitro* mutant differs from it by 2 kcal/mol (it does not matter whether the MFE went down to −7 or went up to −3 kcal/mol). If 700 out of 1000 computer-generated mutants have their MFEs either >−3 or <−7 kcal/mol, it means that for this particular gene a mutant recorded in the *in vitro* experiment changes its MFE to a greater extent than 30% of the randomly mutated sequences.

Suppose that experimentally observed mutations in essential genes lead to bigger MFE changes than only 10% of random mutations, while in the data set of mutations in nonessential genes, MFE changes bigger than those of random mutants are observed in 50% of the cases. This would indicate that the evolutionary constraints acting on mRNA structure in essential genes are stronger that those acting on dispensable genes.

In the *E. coli* genome sampled at 40 000th generation, 523 nucleotide substitutions occurred in 485 genes (11.5% of all *E. coli* genes), of which 48 genes were essential, 348 nonessential and 89 genes either could not be successfully mapped from the REL606 to the MG1655 strain or had unknown essentiality status (Table 1). A great majority of the mutated genes (92.2%) have only one SNP mutation. For the mutated genes, the ratio between the number of essential and nonessential genes is 0.138; while for nonmutated genes, this ratio is 0.206. The latter finding is in agreement with the report by Jordan *et al.* (29) showing that essential genes in bacteria accumulate mutations less frequently than nonessential genes do. The majority of mutations are nonsynonymous (Table 2), with the ratio of synonymous to nonsynonymous mutations in essential genes (0.082) being somewhat lower than in nonessential genes (0.227). The *P*-value calculated using a binomial test (4 synonymous SNPs out of 53 in essential genes *vs* 70 synonymous SNPs out of 379 in nonessential genes) equals 0.048.

**Table 1.** The number of all, essential and nonessential genes in which a particular number of SNPs occurred during the ‘long-term evolution experiment’ between the first and the 40 000th generations

Genes type	Number of mutations per gene			Total
	1	2	3	
All genes^a^	447	36	2	485
Essential genes	43	5	0	48
Nonessential genes	318	29	1	348

^a^Including those with unknown essentiality status.

**Table 2.** The divisions of synonymous and nonsynonymous mutations

Mutation type	All genes	Essential genes	Nonessential genes
Synonymous	83	4	70
Nonsynonymous	442	49	309

**Figure 2.**
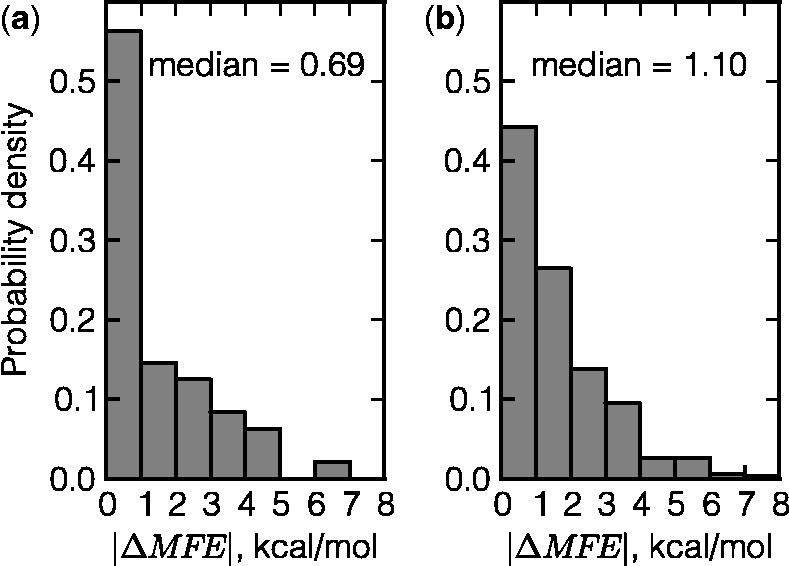
Histograms of absolute changes in MFE values for essential (**a**) and nonessential (**b**) genes.

SNPs cause changes in the MFE and structural perturbations in many, though not all, mRNAs (Table 3). Specifically, 27.1% of essential genes do not change the MFE value, while only 14.7% of nonessential genes demonstrate the same MFE values for both original and mutated mRNAs. In general, SNPs in essential genes change the MFE values (median = 0.69 kcal/mol) to a smaller extent than do mutations in nonessential genes (median = 1.10 kcal/mol) (Figure 2). We compared the properties of essential and nonessential genes that could influence MFE calculations, but found no confounding factors (data not shown). Both groups of genes have the same average GC content. While essential genes tend to be somewhat shorter than nonessential ones (Figure 1), neither in essential nor in nonessential genes do the differences in MFE between the native and mutated sequences depend on mRNA length. Additionally, different types of mutations (e.g. C → G) occur in these two data sets equally often. At the same time, mutations observed at the 40 000th generation *in vitro* are more likely to reduce MFE of nonessential genes than the essential ones. MFE value decreased in 56.0% of the nonessential mutants, while only 45.8% of the essential ones demonstrated MFE reduction. A possible interpretation could be that ancestral essential genes were folded in structures that caused the values of their folding energies to be close to the minimum (robust); in contrast, nonessential genes had MFEs more distant from the minimal values. Thus, mutations were less likely to reduce energies of essential genes.

**Table 3.** The number of essential and nonessential genes that decrease, increase or do not change their MFE value on mutation

Gene type	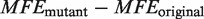			Total
	<0	=0	>0	
Essential	22 (45.8%)	13 (27.1%)	13 (27.1%)	48
Nonessential	195 (56.0%)	51 (14.7%)	102 (29.3%)	348

**Figure 3.**
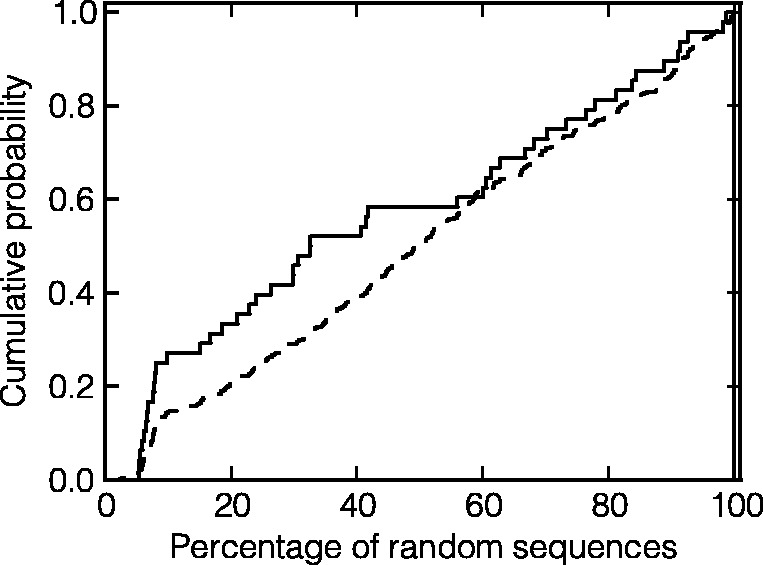
Cumulative distribution functions of the percentages of randomly introduced *in silico* mutations that change the MFE values less than the mutations occurring *in vitro* for essential genes (solid line) and nonessential genes (dashed line). Each curve gives the probability that the MFE change in a particular gene due to an actual mutation will be higher than MFE changes observed in a given percentage of genes with randomly introduced mutations.

We subsequently compared the absolute values of MFE changes caused in each mRNA by naturally occurring and an equal number of randomly introduced synonymous mutations, thus avoiding those mutations in the sequences, generated *in silico*, that could be eliminated by purifying selection due to their effect on the encoded protein. For each mRNA, we determined the fraction of *in silico* derivatives, which change their MFEs less than the mutant observed *in vitro*. These percentages are much lower in essential *E. coli* genes than in nonessential genes (Table 4), implying that mutations accumulated in essential *E. coli* genes affect MFEs (and hence secondary structure) to a lesser extent than mutations in nonessential genes. This effect is further demonstrated by the fact that the cumulative distribution function corresponding to essential genes elevates considerably faster at the beginning (Figure 3). The difference between values for essential and nonessential genes is statistically significant according to the Mann–Whitney U test (*P* = 0.044). Our results suggest that mRNA secondary structure imposes substantially smaller selective pressure at the mutations taking place in nonessential genes because the median of their effect on MFE is 50.2% of what the random set of mutations would cause. By contrast, the median value of how mutations occurring in essential genes influence MFEs is only 32.6% of what random mutations would do.

**Table 4.** Summary of MFE changes in mRNA secondary structures of essential and nonessential genes

Gene type	Number of genes	Lower quartile %	Median %	Upper quartile %
Essential	48	9.4	32.6	71.1
Nonessential	348	25.5	50.2	75.0

Lower quartile, median and upper quartile values are presented for the distributions of percentages of computer-generated mutants with randomly introduced mutations that change the MFE less than the naturally occurring mutations.

## DISCUSSION

The importance of mRNA secondary structure for gene expression was demonstrated for many organisms including bacteria (30–33), human (34,35) and *Drosophila* (36). These studies showed that synonymous SNPs altering mRNA folding may result in decreased mRNA stability and may also change expression efficiency. In a recent *in vitro* study, we introduced mutations into an influenza gene, particularly into a region of the gene encoding for a functionally important protein domain (13). As a result of the perturbations in the RNA structure caused by these mutations, gene expression was significantly reduced. Mutations altering RNA structures thus had a functional knockout effect. We also demonstrated that mutations disruptive to RNA structure may impair transcription without facilitating mRNA degradation. Thus, a new mechanism of viral evolution was proposed (13). We hypothesized that mutations disruptive to RNA structures would likely be eliminated to preserve the gene regions encoding for functionally important sites of viral proteins. Following this line of thought, the goal of the present study was to examine whether preservation of mRNA structures is implicated in the evolution of bacteria.

An important evolutionary characteristic of bacterial genes is their essentiality for organism survival, which can be experimentally assessed based on absence of growth on knockout. We hypothesized that if some of the mutations causing perturbations in mRNA structures also result in reduction in expression levels of bacterial genes, these mutations are more likely to be eliminated by purifying selection if they take place in essential rather than nonessential genes. Indeed, we found that mRNA secondary structures of essential genes are more conserved than those of nonessential genes in bacteria. Previous work revealed that essential bacterial genes are more evolutionarily conserved than nonessential ones (29,37). It was shown that in the *E. coli* genome paired DNA bases have lower propensities to mutate than unpaired bases (38,39). Based on the comparison of the *E. coli* and *Salmonella typhi* genomes, it was concluded that homologous RNAs of polycistronic genes in both organisms have significantly higher folding potential than randomized sequences, which is a sign that natural selection is acting to preserve RNA secondary structure in the coding regions of polycistronic genes (7). However, to the best of our knowledge, preservation of intact mRNA structures of individual genes has not yet been assessed as a potential constraint on the evolution of bacterial genes.

As the best available proxy for *E. coli* B essential genes we used experimentally determined the essentiality status of genes in the closely related *E. coli* K-12 genome (25). Such homology mapping may not always be accurate, even between similar organisms, owing to possible differences in gene regulation, posttranslational modifications and other cellular processes. An additional factor potentially masking the true magnitude of the effect is that we used changes in MFE as an indirect indication that the secondary structure of the mRNA has changed. However, changes in RNA sequence and the resulting perturbations of its structure may in fact take place without causing MFE changes (Table 3). Owing to these obvious limitations, we believe that our results represent a conservative estimate of the role played by mRNA structure in constraining mutations.

Our results point to the preservation of coding mRNA structures as a previously unappreciated factor influencing bacterial evolution. Until now, selective pressure in coding regions was thought to primarily act against mutations that either impair protein function and stability or affect robustness against mistranslations (40). In particular, selection against mistranslation-induced protein misfolding is currently considered to be the major factor determining the strong dependence of protein evolutionary rate on the level of expression (41). The bulk of this research has thus been devoted to the ‘protein half of the equation’—translation, folding and function. In the past few years, attention is being increasingly focused on the noncoding selective pressure in coding regions, which is manifested by the presence of synonymous constraint (42–44). Such noncoding selective pressure may be caused, on one hand, by the presence of various functional elements, such as microRNA binding sites, transcription factor binding sites and splicing enhancers in eukaryotic mRNAs, and, on the other hand, by the formation of RNA structural elements playing a role in mRNA localization, degradation and interactions with other molecules. This article presents the first statistical evidence linking mRNA folding to bacterial evolution. Our principal finding is that purifying selection tends to eliminate those mutations in essential genes that lead to greater changes of MFE values and, therefore, may be more disruptive for the corresponding mRNA secondary structures. This effect is implying that synonymous mutations disrupting mRNA secondary structures may directly affect the fitness of the organism.

## SUPPLEMENTARY DATA

Supplementary Data are available at NAR Online: Supplementary Tables 1–3 and Supplementary Figure 1.

## FUNDING

DFG International Research Training Group ‘Regulation and Evolution of Cellular Systems’ [GRK 1563]; Russian Foundation for Basic Research [RFBR 09-04-92742]. Funding for open access charge: DFG International Research Training Group ‘Regulation and Evolution of Cellular Systems’ [GRK 1563].

*Conflict of interest statement.* None declared.

## Supplementary Material

Supplementary Data
