# Supplementary material for: Conservation of mRNA secondary structures may filter out mutations in Escherichia coli evolution
Source: Nucleic Acids Res. 2013 Jun 19;41(16):7854–60. doi: 10.1093/nar/gkt507 (PMC3763529; doi:10.1093/nar/gkt507)
Supplement: Supplementary Data [file supp_41_16_7854__index.html]

Conservation of mRNA secondary structures may filter out mutations in Escherichia coli evolution — Conservation of mRNA secondary structures may filter out mutations in Escherichia coli evolution — Supplementary Data 

# Conservation of mRNA secondary structures may filter out mutations in *Escherichia coli* evolution

## Supplementary Data

files

**Files in this Data Supplement:**

- Supplementary Data - pdf file
- Supplementary Data - docx file
- Supplementary Data - csv file
- Supplementary Data - csv file
