# Supplementary material for: Conservation of mRNA secondary structures may filter out mutations in Escherichia coli evolution
Source: Nucleic Acids Res. 2013 Jun 19;41(16):7854–60. doi: 10.1093/nar/gkt507 (PMC3763529; doi:10.1093/nar/gkt507)
Supplement: Supplementary Data [file supp_gkt507_nar-00074-r-2013-File005.pdf]

Supplementary Figure S1:

A.

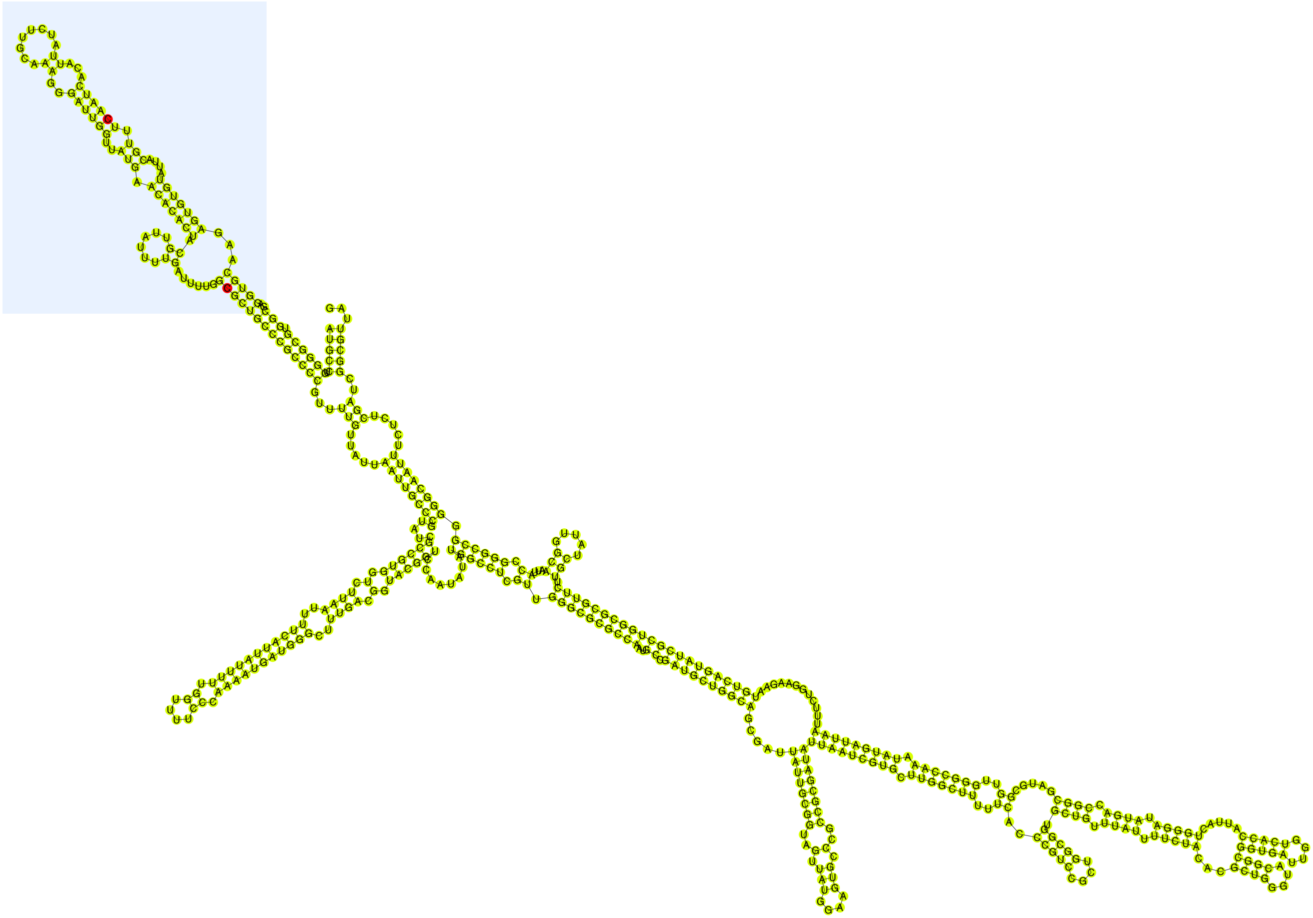

B.

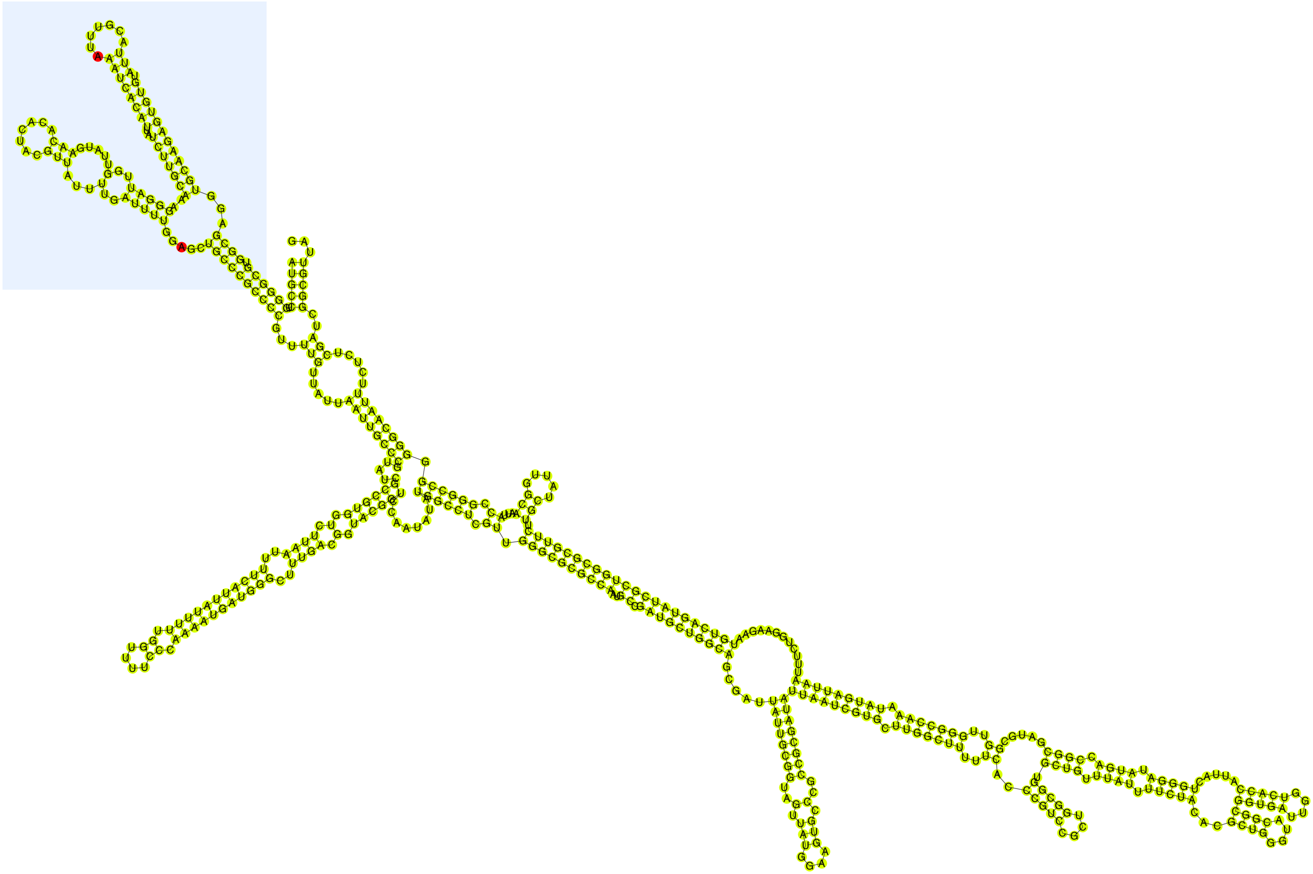

In silico predicted structures of mRNAs for the nonessential *yphA* gene before (A) and after (B) two point mutations (depicted with red color) acquired in the course of the "Long-Term Evolution Experiment". Difference in optimal structures predicted by RNAfold exemplifies perturbations induced by mutations altering minimal free energy of the mRNA ($|\Delta G|=2.9$ kcal/mol). This figure is provided for visualization purposes only and does not represent the entire ensemble of possible RNA structures.
