# Supplementary material for: Conservation of mRNA secondary structures may filter out mutations in Escherichia coli evolution
Source: Nucleic Acids Res. 2013 Jun 19;41(16):7854–60. doi: 10.1093/nar/gkt507 (PMC3763529; doi:10.1093/nar/gkt507)
Supplement: Supplementary Data [file supp_gkt507_nar-00074-r-2013-File006.docx]

Supplementary Table S1: Profile of the occurring mutations in essential and nonessential genes, as well as numbers of mutations of particular type in the background distributions for essential and nonessential genes. Z-test for two proportions was conducted for every type of substitutions between *in silico* introduced mutations for essential and nonessential genes. P-value for every type of substitution is provided in the last column. Also, the ratios of transitions to transversions in the computer-introduced mutations for every gene were calculated and distributions of such ratios between essential and nonessential genes were compared. According to the Mann-Whitney U test, p-value = 0.38.

| Substitution | Essential | Nonessential | Essential random | % | Nonessential random | % | P-value |
| --- | --- | --- | --- | --- | --- | --- | --- |
| C:G -> A:U | 2 | 27 | 6586 | 0.165 | 49103 | 0.163 | 0.38 |
| A:U -> C:G | 46 | 342 | 3526 | 0.088 | 26195 | 0.087 | 0.41 |
| A:U -> U:A | 3 | 0 | 4295 | 0.108 | 32795 | 0.109 | 0.38 |
| C:G -> G:C | 0 | 0 | 5706 | 0.143 | 43415 | 0.144 | 0.45 |
| C:G -> U:A | 2 | 8 | 11064 | 0.277 | 82388 | 0.274 | 0.17 |
| A:U -> G:C | 0 | 2 | 8738 | 0.219 | 66845 | 0.222 | 0.13 |
| Total | 53 | 379 | 39915 | 1.0 | 300741 | 1.0 |  |
